# Lifespan Trajectories of Asymmetry in White Matter Tracts

**DOI:** 10.1002/hbm.70519

**Published:** 2026-06-06

**Authors:** Sam Bogdanov, Praitayini Kanakaraj, Michael E. Kim, Jessica Samir, Chenyu Gao, Karthik Ramadass, Gaurav Rudravaram, Nancy R. Newlin, Derek Archer, Timothy J. Hohman, Angela L. Jefferson, Victoria L. Morgan, Alexandra Roche, Dario J. Englot, Susan M. Resnick, Lori L. Beason Held, Laurie E. Cutting, Laura A. Barquero, Micah A. D'Archangel, Tin Q. Nguyen, Kathryn L. Humphreys, Yanbin Niu, Sophia Vinci‐Booher, Carissa J. Cascio, The HABS‐HD Study Team, Alzheimer's Disease Neuroimaging Initiative, The BIOCARD Study Team, Sid E O'Bryant, Kristine Yaffe, Arthur Toga, Robert Rissman, Leigh Johnson, Meredith Braskie, Kevin King, James R Hall, Melissa Petersen, Raymond Palmer, Robert Barber, Yonggang Shi, Fan Zhang, Rajesh Nandy, Roderick McColl, David Mason, Bradley Christian, Nicole Phillips, Stephanie Large, Joe Lee, Badri Vardarajan, Monica Rivera Mindt, Amrita Cheema, Lisa Barnes, Mark Mapstone, Annie Cohen, Amy Kind, Ozioma Okonkwo, Raul Vintimilla, Zhengyang Zhou, Michael Donohue, Rema Raman, Matthew Borzage, Michelle Mielke, Beau Ances, Ganesh Babulal, Jorge Llibre‐Guerra, Carl Hill, Rocky Vig, Zhiyuan Li, Simon N. Vandekar, Panpan Zhang, John C. Gore, Stephanie J. Forkel, Bennett A. Landman, Kurt G. Schilling

**Affiliations:** ^1^ Medical Scientist Training Program Vanderbilt University Nashville Tennessee USA; ^2^ Department of Computer Science Vanderbilt University Nashville Tennessee USA; ^3^ Department of Psychological Science and Neuroscience Belmont University Nashville Tennessee USA; ^4^ Department of Electrical and Computer Engineering Vanderbilt University Nashville Tennessee USA; ^5^ Vanderbilt Memory and Alzheimer's Center Vanderbilt University Medical Center Nashville Tennessee USA; ^6^ Vanderbilt Genetics Institute Vanderbilt University Medical Center Nashville Tennessee USA; ^7^ Vanderbilt Brain Institute Vanderbilt University Nashville Tennessee USA; ^8^ Department of Medicine Vanderbilt University Medical Center Nashville Tennessee USA; ^9^ Department of Neurology Vanderbilt University Medical Center Nashville Tennessee USA; ^10^ Department of Psychology and Human Development Vanderbilt University Nashville Tennessee USA; ^11^ Department of Psychiatry and Behavioral Sciences Vanderbilt University Medical Center Nashville Tennessee USA; ^12^ Department of Radiology and Radiological Sciences Vanderbilt University Medical Center Nashville Tennessee USA; ^13^ Vanderbilt University Institute of Imaging Science Nashville Tennessee USA; ^14^ Department of Biomedical Engineering Vanderbilt University Nashville Tennessee USA; ^15^ Department of Neurological Surgery Vanderbilt University Medical Center Nashville Tennessee USA; ^16^ Laboratory of Behavioral Neuroscience National Institute on Aging, National Institutes of Health Baltimore Maryland USA; ^17^ Department of Special Education Peabody College of Education and Human Development Nashville Tennessee USA; ^18^ Life Span Institute and Department of Psychology University of Kansas Lawrence Kansas USA; ^19^ Department of Computer Science Park University Parkville Missouri USA; ^20^ Department of Biostatistics Vanderbilt University Medical Center Nashville Tennessee USA; ^21^ Donders Centre for Cognition Radboud University Nijmegen the Netherlands; ^22^ Max Planck Institute for Psycholinguistics Nijmegen the Netherlands; ^23^ Brain Connectivity and Behaviour Laboratory Paris France

**Keywords:** asymmetry, diffusion MRI, lateralization, lifespan development, tractography, white matter

## Abstract

Asymmetry in white matter is believed to give rise to the brain's capacity for specialized processing and is involved in the lateralization of various cognitive processes, such as language and visuo‐spatial reasoning. Although studies of white matter asymmetry have been previously documented, they have often been constrained by limited age ranges, sample sizes, or the scope of the tracts and structural features examined. While normative lifespan charts for brain structures are emerging, comprehensive charts detailing white matter asymmetries across numerous pathways and diverse structural measures have been notably absent. This study addresses this gap by leveraging a large‐scale dataset of 35,120 typically developing and aging individuals, ranging from 0 to 100 years of age, from 50 primary neuroimaging studies. We generated comprehensive lifespan trajectories for 30 lateralized association and projection white matter tracts, examining six distinct microstructural and macrostructural features of these pathways. Our findings reveal that: (1) asymmetries are widespread across the brain's white matter and are present in all 30 pathways; (2) for a given pathway, the degree and direction of asymmetry differ between features of tissue microstructure and pathway macrostructure; (3) asymmetries vary across and within pathway types (association and projection tracts); and (4) these asymmetries are not static, following unique trajectories across the lifespan, with distinct changes during development, and a general trend of becoming more asymmetric with increasing age (particularly in later adulthood) across pathways. This study represents the most extensive characterization of white matter asymmetry across the lifespan to date, charting how lateralization patterns emerge, mature, and change throughout life. It provides a foundational resource for understanding the principles of white matter organization from early to late life, its relation to functional specialization and inter‐individual variability, and offers a key reference for interpreting deviations during healthy development and aging as well as those associated with clinical populations.

## Graphical abstract

(A) Large‐scale lifespan modeling of white matter asymmetry. Diffusion MRI data from 35,000+ individuals (0‐100 years) across 50 cohorts were used to generate normative lifespan trajectories of white matter asymmetry. Thirty bilateral tracts were segmented, and microstructural (FA, MD, AD, RD) and macrostructural (volume, length) features were extracted. A lateralization index (LI) was computed for each tract‐feature pair and modeled using a centile‐based normative framework (GAMLSS), enabling estimation of age‐specific population distributions. (B) Lateralization differs by tract and structural feature. Asymmetry was widespread but highly specific to tract and feature. Microstructural measures exhibited smaller magnitude asymmetries, whereas macrostructural features showed larger effects and greater inter‐individual variability. Individual tracts frequently demonstrated opposing asymmetry directions across features, underscoring distinct biological contributions of tissue microstructure and pathway geometry. Population‐level patterns ranged from strong, consistent lateralization to substantial heterogeneity within age groups. (C) Asymmetry is dynamic across the lifespan. White matter asymmetry varied continuously across development and aging, with tract‐ and feature‐specific trajectories. Patterns included early establishment, gradual strengthening or attenuation, and occasional direction reversals. Variability was greatest in early life and older adulthood, and many pathways showed increasing asymmetry magnitude with age. These results demonstrate that asymmetry is a dynamic property of white matter organization across the lifespan.

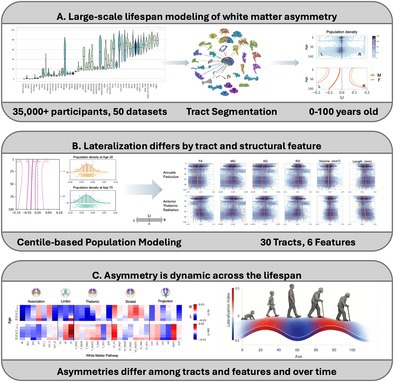

## 1 Introduction

Brain asymmetry is an organizing principle of the nervous system that enables functional specialization (Ocklenburg et al. 2024). Nearly two centuries ago, the pioneering clinical observations of Marc Dax (1836) (Dax 1865; Manning and Thomas‐Anterion 2011) and later Paul Broca (1860s) (Amunts et al. 1999; Broca 1861) first linked left‐hemisphere lesions to specific language deficits, establishing that complex cognitive functions are frequently lateralized to one hemisphere. This hemispheric division of labor is supported by an underlying structural architecture that enhances neural efficiency, allowing for parallel processing of different computations while minimizing cross‐hemispheric conduction delays (Brincat and Miller 2025). Asymmetries in functions as diverse as language (Bishop 2013), visuospatial attention (Thiebaut de Schotten, Dell'Acqua, et al. 2011), and motor control (Callaert et al. 2011) are thought to depend on this lateralized brain circuitry.

The structural substrate of this functional specialization is the brain's white matter connectome (Biswal et al. 2010; Sporns et al. 2005). Modern neuroimaging—specifically, diffusion‐weighted MRI (dMRI) in combination with fiber tractography (Jeurissen et al. 2019)—enables a noninvasive “virtual dissection” to segment and study the macrostructure of these pathways, as well as a “virtual histology” to probe their tissue microstructure (Alexander et al. 2019). Macrostructural measures describe morphometric features of pathway size and geometry, including volume, lengths, and areas (Yeh 2020). In contrast, microstructural measures are sensitive to cellular‐level features including axonal coherence, packing density, and myelination (Beaulieu 2002). Characterizing white matter asymmetry at both macro‐ and microstructure scales is essential, as they capture distinct biological properties that can follow different trajectories across development and aging.

Many diffusion MRI studies have confirmed robust hemispheric asymmetries in specific white matter tracts, mirroring classic left–right differences in brain function (Dulyan et al. 2025). In particular, the arcuate fasciculus—a key frontotemporal language pathway—is typically larger or more developed in the left hemisphere, consistent with left‐hemisphere dominance for language (Andrulyte et al. 2024). This leftward asymmetry of the arcuate is functionally significant: individuals with a more pronounced leftward arcuate show stronger left‐hemisphere activation during speech processing tasks (Ocklenburg et al. 2013; Propper et al. 2010), and higher performance in verbal tasks (Catani et al. 2007). Conversely, frontoparietal pathways associated with attention tend to be right‐lateralized—for example, the inferior branch of the superior longitudinal fasciculus (SLF‐III) is consistently larger on the right (Amemiya et al. 2021; Howells et al. 2018; Thiebaut de Schotten, Dell'Acqua, et al. 2011), aligning with the right hemisphere's specialization in spatial attention. Notably, these tract‐level asymmetries emerge by early childhood and are thought to scaffold normal cognitive development (Ghasoub et al. 2025; Marcelle et al. 2025) (e.g., supporting mature language networks). Further, atypical patterns of white matter laterality have been linked to clinical conditions, with altered or atypical (or even reversed) patterns of white matter asymmetry reported in schizophrenia (Gomez‐Gastiasoro et al. 2019; Ho et al. 2017), autism spectrum disorder (Carper et al. 2016), dyslexia (Banfi et al. 2019; Rimrodt et al. 2010), and Parkinson's disease (Chen et al. 2022), among others (Saltoun et al. 2025).

Despite this growing body of work (Carper et al. 2016; Demnitz et al. 2021; Hau et al. 2017; Honnedevasthana Arun et al. 2021; Kumpulainen et al. 2023; Mundorf et al. 2021; Roe et al. 2023; Shu et al. 2021; Shu et al. 2015; Takao et al. 2013; Zhu et al. 2023), current knowledge of white matter asymmetry rests largely on static snapshots from studies with modest sample sizes, narrow age ranges, and limited pathway and feature coverage. This has led to a fragmented literature in which the reported laterality for a given pathway often diverges, due to variations in sample size, age range/cohorts, and the specific structural feature(s) investigated. Moreover, most reports simply focus on testing whether the group mean differs from zero, rather than describing the age‐specific distribution and how it changes across development and aging. This leaves unanswered questions about when asymmetry emerges, whether it strengthens or weakens at different life stages, and how widely it varies among healthy individuals at a given age. In short, the field lacks a lifespan‐wide normative reference that quantifies the distribution of tract‐level asymmetry across development and aging.

Here, we assemble 35,120 scans from 50 population‐based cohorts spanning 0–100 years and construct age‐varying brain asymmetry charts for white matter pathways. Rather than centering on a binary test of whether asymmetry exists, we use an estimation framework to quantify the *magnitude*, *direction*, and *age‐specific distribution* of asymmetry. Specifically, we: (1) establish normative lifespan trajectories for all investigated features and pathways; (2) identify which tracts and features exhibit asymmetry during key periods of development, adulthood, and aging; (3) quantify the population‐level distribution of right–left asymmetries within these periods; (4) determine whether—and at what ages—the direction of asymmetry reverses; and (5) assess how the magnitude of asymmetry changes dynamically (i.e., increases or decreases) across infancy, development, and later life. A priori, we anticipate that normative modeling would reveal structured, tract‐ and feature‐specific distributions of laterality that change across the lifespan, including shifts in both the median and the spread of asymmetry across development and aging. Together, these charts provide a population reference to contextualize individuals and cohorts, enabling future developmental and clinical studies to interpret asymmetry in an age‐appropriate framework.

## 2 Materials and Methods

**FIGURE 1:**
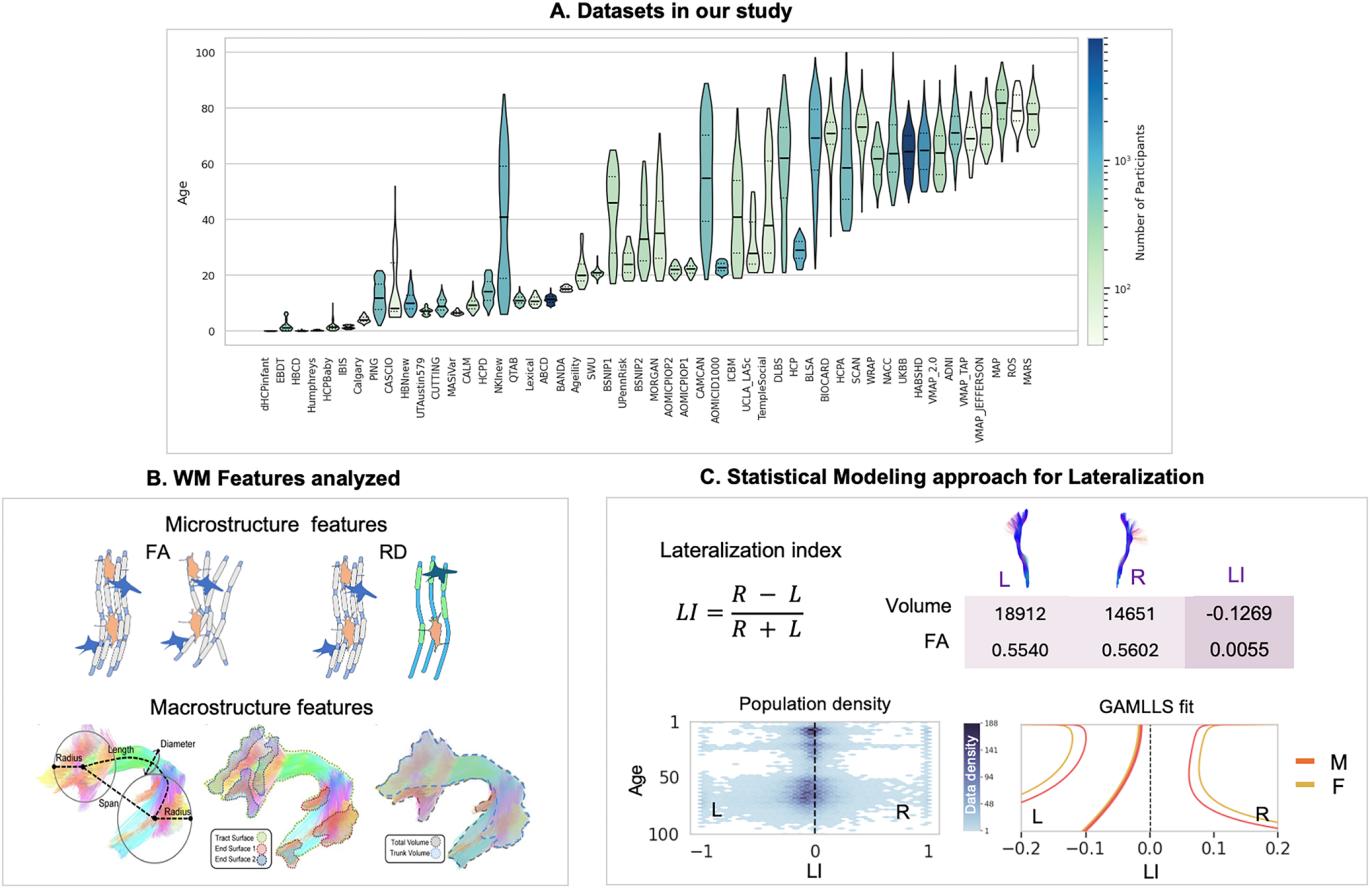
Overview of the study datasets, white matter features, and analytical framework. (A) Age distributions for each of the 50 contributing datasets (violin plots), illustrating broad coverage from 0 to 100 years. Color encodes the number of participants per dataset (log scale). (B) Features extracted for each of the 30 bilateral pathways. Microstructural indices (e.g., Fractional Anisotropy, and Mean, Axial and Radial diffusivities; FA, MD, AD, and RD) summarize tissue organization and axonal/myelin density; macrostructural indices (e.g., tract volume and length) capture pathway size and geometry. Macrostructural cartoon reproduced under CC‐BY from Yeh 2020. (C) Analysis pipeline. For each participant, white matter pathways were segmented, and features were extracted. A Lateralization Index (LI) was calculated for each tract‐feature pair. These LIs were used as input for a normative modeling framework (GAMLSS) to generate age‐specific population centile curves.

We analyzed 35,120 diffusion MRI scans from 50 population‐based cohorts spanning 0–100 years (Figure 1A). For each participant, we derived tract‐level measures for 30 bilateral white matter pathways, capturing both microstructure (diffusion tensor imaging indices reflecting tissue organization, axonal density, and myelination) and macrostructure (tract volume and length) (Figure 1B). We computed a laterality index for every tract‐feature pair (Figure 1C) and modeled age‐varying centile curves to estimate the population distribution (i.e., typically showing 2.5th–25th–50th–75th–97.5th centiles) at each age. Our analyses summarize patterns across the developmental and aging windows, quantify inter‐individual variability, and identify tracts and features with asymmetry or direction reversals.

We note that we emphasize and describe population *centiles*—which describe the expected range of individual values at a given age—rather than *confidence intervals*, which only quantify the precision of the median estimate; we therefore do not perform null‐hypothesis significance tests, which are trivially positive at very large N. Study cohorts, image preprocessing, white matter pathway segmentation, statistical analysis and normative modeling are described below.

### 2.1 Participants and Study Cohorts

The final dataset comprised 35,120 cross‐sectional diffusion MRI scans aggregated from 50 independent, population‐based cohorts (Figure 1A; see Table S1 for a detailed description of each cohort). To ensure a cross‐sectional analysis, only the earliest available scan was included for any participant with longitudinal data, so that each individual is represented only once. The cohorts consist of typically developing and aging participants with broad geographic representation. Studies were included that contained broad patient populations; for these studies, only neurotypical participants without a pathologic diagnosis (as defined by each cohort) were included in the analyses performed in this study. Acquisition parameters varied across studies (see Table S2 for detailed description of voxel sizes and b‐values), necessitating downstream harmonization—including both standardized preprocessing (see Section 2.2), uniform tract segmentation (see Section 2.3.1), and statistical modeling within the lifespan (see Section 2.4.2). Demographic information was harmonized across studies where available, including sex (53.7% female) and handedness (of 14,220 participants with handedness data, 9.3% were left‐handed). In studies with handedness available, self‐report or a questionnaire (typically the Edinburgh Handedness Inventory) was used to determine handedness (handedness experiments are given as Supporting Information). Cognitive and behavioral measures were not consistently available across the consortium and were therefore not included in the present analysis. All contributing studies received ethical approval from their local institutional review boards.

### 2.2 Diffusion MRI Processing Pipeline

All diffusion MRI data were processed with a single, standardized workflow using the PreQual pipeline (v 1.0.8) applied identically across cohorts to promote reproducibility (Cai et al. 2021). This pipeline corrected for susceptibility‐induced EPI distortions, head motion, and eddy current artifacts. To ensure consistency and reproducibility across the 50 cohorts, which varied in scanner and acquisition parameters, the same pipeline version was applied to all datasets, with specific flags adapted for each study's raw data structure. Following this preprocessing, diffusion tensor imaging (DTI) models were fitted to generate voxel‐wise maps of fractional anisotropy (FA), mean diffusivity (MD), axial diffusivity (AD), and radial diffusivity (RD). Quality control was conducted at two stages (Kim, Gao, et al. 2024; Kim, Ramadass, et al. 2024). First, automated logs and visual reports from PreQual were reviewed to identify corruption, gross motion, or failed corrections. Second, the derived scalar maps were visually inspected to verify anatomical plausibility and artifact mitigation. Scans failing QC at either stage were excluded prior to downstream analyses.

### 2.3 White Matter Pathway Segmentation and Feature Extraction

#### 2.3.1 Tract Segmentation

The preprocessed dMRI data for each participant were upsampled to 1 mm isotropic resolution and input into TractSeg (v2.8) (Wasserthal et al. 2018), a convolutional neural network (CNN) framework for white matter bundle segmentation (Wasserthal et al. 2018). TractSeg operates directly on local fiber orientation images, segmenting tracts without requiring whole‐brain tractography or inter‐subject registration. We employed the TractSeg pipeline with default parameters, which uses the MRtrix3 implementation of Constrained Spherical Deconvolution (CSD) to extract fiber orientation peaks from the DWI data for downstream bundle segmentation, bundle endpoints segmentation, tract‐orientation mapping, and subsequent tractography. The pre‐trained model automatically segmented 72 white matter bundles per participant. From these, we selected 60 lateral pathways (i.e., 30 bilateral tract pairs), excluding midline commissural structures, for asymmetry analyses. The set spans association and projection systems (with limbic, thalamic, and striatal subdivisions noted in Table 1).

**TABLE 1:** List of 30 bilateral white matter tracts included in asymmetry analysis.

Pathways	Subcategory	Tract	Acronym
Association	Association	Arcuate Fasciculus	AF
		Middle Longitudinal Fasciculus	MLF
		Inferior Fronto‐Occipital Fasciculus	IFOF
		Inferior Longitudinal Fasciculus	ILF
		Superior Longitudinal Fasciculus I	SLF_I
		Superior Longitudinal Fasciculus II	SLF_II
		Superior Longitudinal Fasciculus III	SLF_III
	Limbic	Cingulum Bundle	CG
		Uncinate Fasciculus	UF
		Fornix	FX
Projection	Thalamic	Thalamo‐prefrontal	T_PREF
		Thalamo‐premotor	T_PREM
		Thalamo‐precentral	T_PREC
		Thalamo‐postcentral	T_POSTC
		Thalamo‐parietal	T_PAR
		Thalamo‐occipital	T_OCC
		Anterior Thalamic Radiation	ATR
	Striatal	Striato‐fronto‐orbital	ST_FO
		Striato‐prefrontal	ST_PREF
		Striato‐premotor	ST_PREM
		Striato‐precentral	ST_PREC
		Striato‐postcentral	ST_POSTC
		Striato‐parietal	ST_PAR
		Striato‐occipital	ST_OCC
	Projection	Corticospinal Tract	CST
		Frontal Pontine Tract	FPT
		Inferior Cerebellar Peduncle	ICP
		Optic Radiation	OR
		Parieto‐Occipital Pontine Tract	POPT
		Superior Cerebellar Peduncle	SCP

*Note:* As defined by the preset anatomical labels and segmentation outputs of the automated tractography tool TractSeg.

#### 2.3.2 Microstructural and Macrostructural Feature Definition

For each of the 30 segmented tract pairs, we computed two classes of features using the scilpy library (Sherbrooke Connectivity Imaging Lab's open‐source toolkit, version 1.5.0) (scilus/scilpy 2012) (Figure 1B).

Microstructural Features: We calculated four standard DTI indices that reflect cellular‐level tissue properties (Beaulieu 2002; Wheeler‐Kingshott and Cercignani 2009). To derive a single value for each tract, we computed a weighted average of the metric across all voxels within the tract's mask, with the contribution of each voxel weighted by the number of streamlines passing through it.

- Fractional Anisotropy (FA): an index of directional coherence of diffusion, influenced by axonal organization/packing and myelin sheaths.
- Mean Diffusivity (MD): the average magnitude of water diffusion, sensitive to overall water mobility within tissue, influenced by axonal/myelin densities.
- Axial Diffusivity (AD): magnitude of water diffusion parallel to the principal fiber direction, sensitive to the intra‐axonal space and changes in axonal caliber and architecture.
- Radial Diffusivity (RD): magnitude of water diffusion perpendicular to the principal fiber direction. As myelin sheaths are a primary barrier to perpendicular diffusion, increased RD is often interpreted as reflecting demyelination or reduced axonal packing.

Macrostructural Features: We calculated ten features describing the large‐scale geometry and morphology of each tract, of which we primarily focused on two (Schilling et al. 2023; Yeh 2020):

- Tract Volume: The total volume in mm^3^ occupied by the pathway.
- Mean Streamline Length: The average length in mm of the streamlines constituting the tract, reflecting the trajectory extent.

### 2.4 Statistical Analysis and Normative Modeling

#### 2.4.1 Calculation of the Lateralization Index (LI)

For each micro‐ and macrostructural feature, we calculated a *Lateralization Index* (LI) to quantify the degree of asymmetry between the left (L) and right (R) hemispheres for each tract (Figure 1C). We used the formula $LI=\frac{R-L}{R+L}$. This index is bounded between −1 and 1. An LI of 0 indicates perfect symmetry. For this study, positive LI values indicate a rightward asymmetry (i.e., a higher value in the right‐hemisphere tract), while negative LI values indicate a leftward asymmetry (a higher value in the left‐hemisphere tract) (Savic and Lindström 2008). This definition (and data presentation) of the lateralization index is consistent with the expert consensus provided in the *Laterality Indices Consensus Initiative* (Vingerhoets et al. 2023).

#### 2.4.2 Lifespan Normative Modeling Framework

We created lifespan charts of white matter asymmetry using a normative modeling approach. In the context of this large‐scale dataset (*N* > 35,000), traditional null‐hypothesis significance testing is not informative, as even biologically negligible asymmetries would yield a statistically significant result. Our goal was therefore not to ask the binary question of *whether* asymmetry exists, but rather to adopt an estimation framework to describe the full, age‐specific distribution of the LI. This approach, analogous to the creation of pediatric growth charts (Grummer‐Strawn, Reinold, Krebs, Centers for Disease, and Prevention 2010; Santoni et al. 2020), allows for the characterization of the typical range of inter‐individual variation at any given age (Bethlehem et al. 2022).

To model these complex, non‐linear lifespan trajectories, we employed Generalized Additive Models for Location, Scale, and Shape (GAMLSS), implemented in R using the gamlss package (Bethlehem et al. 2022; Rigby and Stasinopoulos 2005). GAMLSS is highly suited for lifespan data as it flexibly models how the entire distribution of the LI—including its median (location, μ), variability (scale, σ), and shape—changes as a non‐linear function of age.

Diffusion MRI measures can vary systematically across scanners and acquisition protocols in multi‐cohort datasets (Grech‐Sollars et al. 2015; Schilling, Rheault, Petit, Hansen, et al. 2021; Schilling, Tax, Rheault, Hansen, et al. 2021). To mitigate these effects, we modeled study cohort as a dataset‐specific effect on both the location and scale of the LI distribution as in Bethlehem et al. (2022), allowing cohort‐level offsets and dispersion differences while estimating a shared age‐varying trajectory. In addition, the LI is a within‐subject normalized ratio, which reduces sensitivity to global scaling differences that affect left and right measurements similarly within an individual.

For each of the 30 tracts and 6 features, a separate GAMLSS model was fit. We modeled the LI as a response variable following a normal distribution (Savic and Lindström 2008), where both the mean (*μ*) and the standard deviation (*σ*) were modeled as functions of age, sex, and study cohort:

(1) $$\mu =f_{\mu }\left(\mathrm{Age}\right)+\beta_{\mu ,\mathrm{sex}}\;\cdot \;\mathrm{Sex}+u_{\mu ,\text{dataset}}$$

(2) $$\begin{array}{c} \log\left(\sigma \right)=f_{\sigma }\left(\mathrm{Age}\right)+\beta_{\sigma ,\mathrm{sex}}\;\cdot \;\mathrm{Sex}+u_{\sigma ,\text{dataset}}\; \end{array}$$

Here, fμ(·) and fσ(·) are smooth functions of age, sex is a fixed effect, and dataset random effects (*u*_μ_, *u*_σ_) capture residual between‐study variation. Allowing σ to vary with age accommodates age‐dependent heteroscedasticity.

Age effects were represented with fractional polynomial (FP) smooths (order up to 3; powers drawn from [−2, −1, −0.5, 0, 0.5, 1, 2, 3] with logarithmic terms for repeated powers). For each tract–feature we fit candidate models with different FP choices for *μ* and *σ* and selected the specification with the lowest Bayesian Information Criterion (BIC) (Bethlehem et al. 2022).

From the parameters of the best‐fitting model, we generated smooth centile curves (e.g., 2.5th, 25th, 50th, 75th, and 97.5th percentiles) across a dense age grid, forming the final normative brain asymmetry charts (Figure 1C).

### 2.5 Characterizing Lifespan Dynamics

To examine white matter asymmetry across the lifespan, we used the normative LI trajectories derived from our GAMLSS models and summarized them using six predefined lifespan epochs: early childhood (2–5 years), childhood (5–12 years), adolescence (12–20 years), early adulthood (20–40 years), midlife (40–60 years), and older adulthood (60–100 years). For analyses requiring discrete “snapshot” summaries, we selected six representative ages corresponding to the median age within each epoch (3, 9, 17, 30, 50, and 80 years). For window‐based analyses, we quantified trends within each epoch directly.

1. What are the characteristics of exemplar lifespan asymmetry curves?

To answer this, we visualized the full, sex‐specific centile curves (2.5th to 97.5th percentile) for selected white matter tracts and features to illustrate general patterns of development, aging, and inter‐individual variability.

2 Which features and pathways exhibit population‐level asymmetry at critical lifespan stages?

We summarized tract‐ and feature‐specific asymmetries at six representative ages (3, 9, 17, 30, 50, and 80 years) using heatmaps that display both the population median LI and the proportion of individuals with rightward vs. leftward lateralization.

3 Do asymmetries reverse direction across the lifespan?

We identified shifts in hemispheric dominance by assessing zero‐crossings in the median LI trajectories, which indicate if and when the direction of population‐level asymmetry changed (e.g., from leftward to rightward, or vice versa).

4 How does the magnitude of asymmetry evolve during different life stages?

Within each of the six lifespan epochs, we quantified the rate of change in asymmetry magnitude by calculating the mean slope of the absolute value of the median LI curve. This allowed us to determine whether the *magnitude of asymmetry* was increasing (strengthening) or decreasing (attenuating) during that life stage.

## 3 Results

We constructed normative lifespan charts of white matter asymmetry by applying our statistical modeling framework to 35,120 individuals spanning 0–100 years. These charts characterize the magnitude, direction, and population‐level variability for 6 micro‐ and macrostructural features across 30 bilateral white matter pathways. This comprehensive analysis revealed several key principles of brain lateralization, which are detailed in the following sections. We begin by illustrating the modeling approach and showing exemplar trajectories that highlight distinct patterns of development and aging.

### 3.1 What Are the Characteristics of Exemplar Lifespan Asymmetry Curves?

**FIGURE 2:**
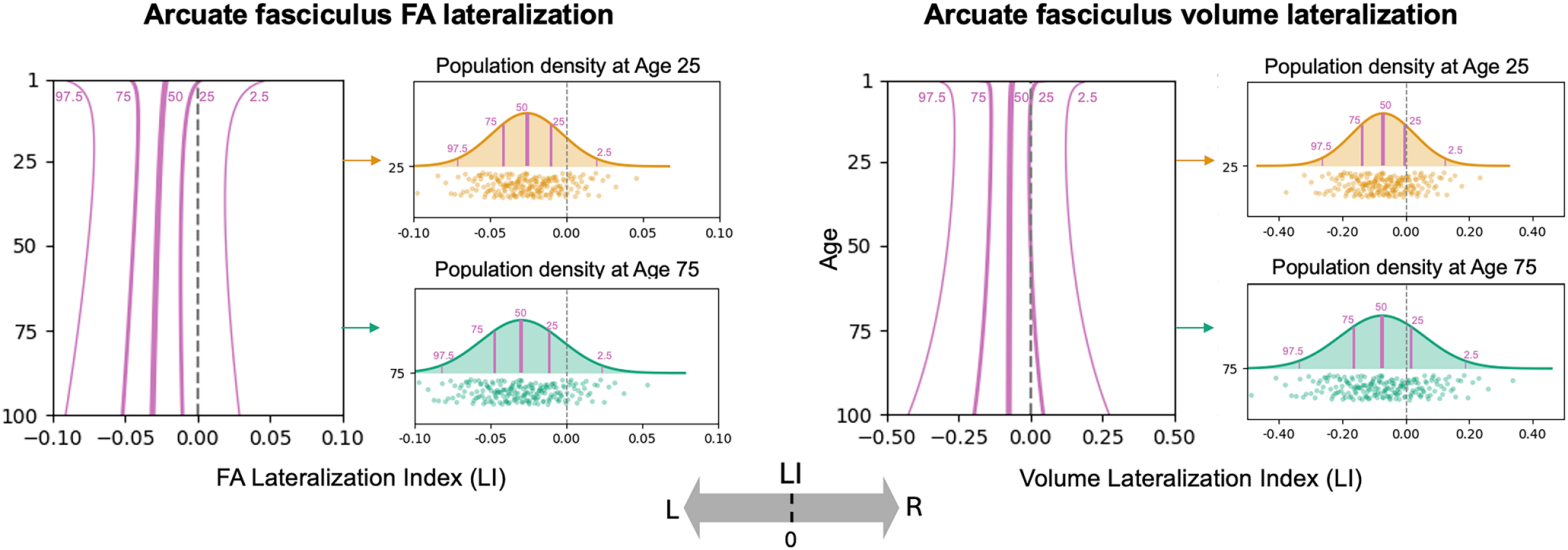
Exemplar asymmetry lifespan curves for the arcuate fasciculus (AF). Lifespan trajectories for fractional anisotropy (FA; a microstructural feature) and tract volume (a macrostructural feature) are shown. Lines represent the 2.5th, 25th, 50th (median), 75th, and 97.5th centile curves, illustrating the full population distribution of the Lateralization Index (LI) across the lifespan. Negative values indicate leftward asymmetry; positive values indicate rightward asymmetry.

The normative lifespan charts revealed that white matter tracts exhibit highly heterogeneous and dynamic patterns of asymmetry, which varied substantially across different pathways, features, and ages. To orient the reader to these results, Figure 2 illustrates the full centile‐based lifespan trajectory for two features of the arcuate fasciculus (AF)—the FA and total volume. These charts depict the population median (50th percentile) and the spread of the population (from the 2.5th to the 97.5th percentile), with lateralization intuitively shown left‐to‐right along the x‐axis, providing a comprehensive view of both the central tendency and the inter‐individual variability in asymmetry at any given age.

**FIGURE 3:**
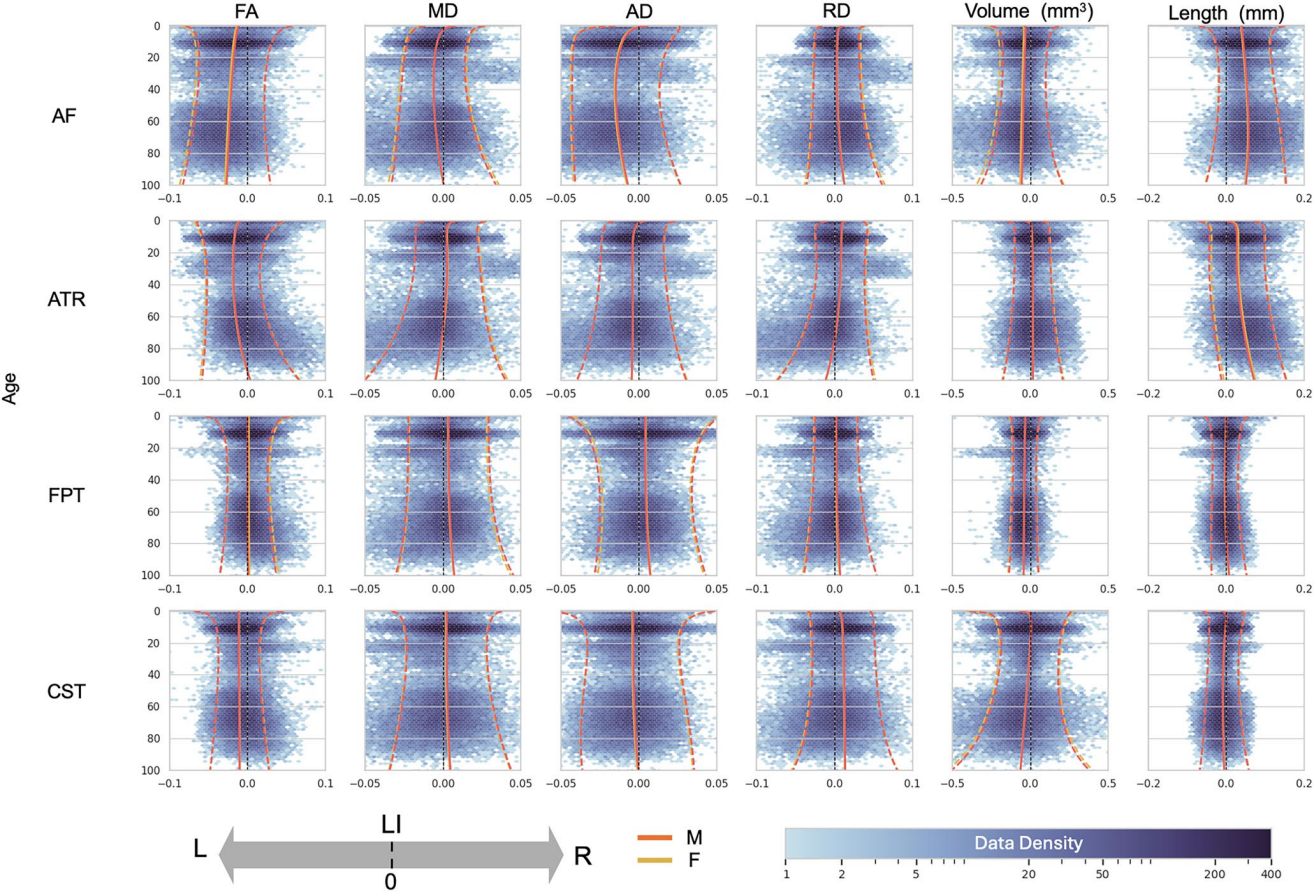
Diverse patterns of lifespan asymmetry across tracts and features. Lifespan trajectories of the LI are shown for four white matter tracts (arcuate fasciculus (AF), anterior thalamic radiation (ATR), frontal pontine tract (FPT), and corticospinal tract (CST)) and six features (FA, MD, AD, RD, volume, and length). Solid lines represent the population median (50th percentile), with the lighter bands showing the interquartile range (25th‐75th percentiles). Red and yellow lines correspond to males and females, respectively. The number of participants along the lifespan that were used to generate the curves is overlaid (log scale). The plots highlight that the magnitude, direction, and age‐related dynamics of asymmetry are highly specific to the tract and feature being measured. Full charts for all tracts and features are provided in Figure S2.

Building on this, Figure 3 displays a broader selection of exemplar trajectories for four well‐studied pathways (AF, Anterior Thalamic Radiation [ATR], Frontal Pontine Tract [FPT], Corticospinal Tract [CST]) across six key micro‐ and macrostructural (FA, MD, AD, RD, tract volume, and tract length) features. Analysis of asymmetry in these same pathways and features according to handedness was also performed (Figure S1).

These charts highlight several overarching principles: First, while asymmetry is common, its magnitude varies by feature type. Microstructural asymmetries were often subtle, with the bulk of the population having LI values between −0.05 and +0.05. For example, the AF showed a consistent leftward FA asymmetry with a population median LI ranging from −0.02 to −0.03 across age and 25th/75th centiles from +0.05 to −0.1. In contrast, macrostructural features exhibited a much wider range of individual variability, often spanning the range from −0.2 to +0.2, with larger median effects (e.g., median LI for ATR length ~0.05; FPT volume ~−0.07). Second, patterns were highly dependent on the combination of tract and metric, where pathways can show opposite lateralization directions across features, highlighting the need to study both micro‐ and macrostructure jointly. For example, the AF shows left‐lateralization for FA and volume but right‐lateralization for RD and length, or the ATR showing general leftward asymmetries for diffusivities and rightward volume and length asymmetries. Third, these asymmetries were not static but changed dynamically across the lifespan, with many tracts showing age‐dependent increases or decreases in lateralization. Fourth, despite these complex dynamics, male and female median trajectories showed general overlap across nearly all tracts and features, suggesting minimal sex differences in the average patterns of asymmetry. Finally, inter‐individual variability was not constant, with wider centile bands in early childhood and older age suggesting a greater diversity of asymmetry patterns during these life stages.

All individual lifespan charts for all 30 tracts and 6 features are provided in Figure S2.

### 3.2 Which Features and Pathways Exhibit Population‐Level Asymmetry at Critical Lifespan Stages?

**FIGURE 4:**
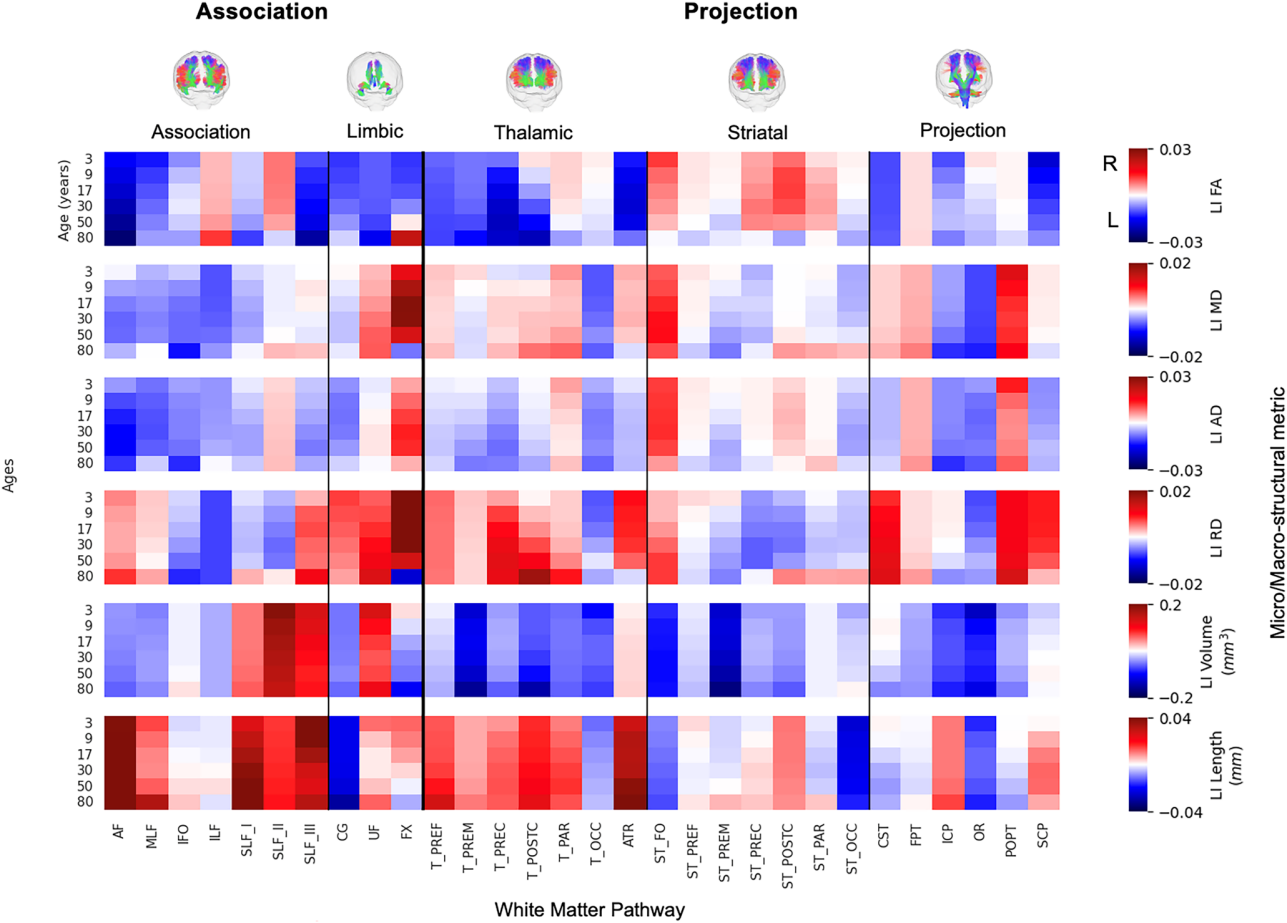
Population‐level asymmetry at key lifespan milestones is highly specific to anatomical pathway and structural feature. Heatmaps depict the population‐median Lateralization Index (LI) for six key features at six discrete age points (3, 9, 17, 30, 50, and 80 years) across 30 major white matter tracts. The color scale reflects the direction and magnitude of the median asymmetry (red: Rightward; blue: Leftward), highlighting that distinct patterns of lateralization are already established in early life and continue to evolve across the lifespan.

**FIGURE 5:**
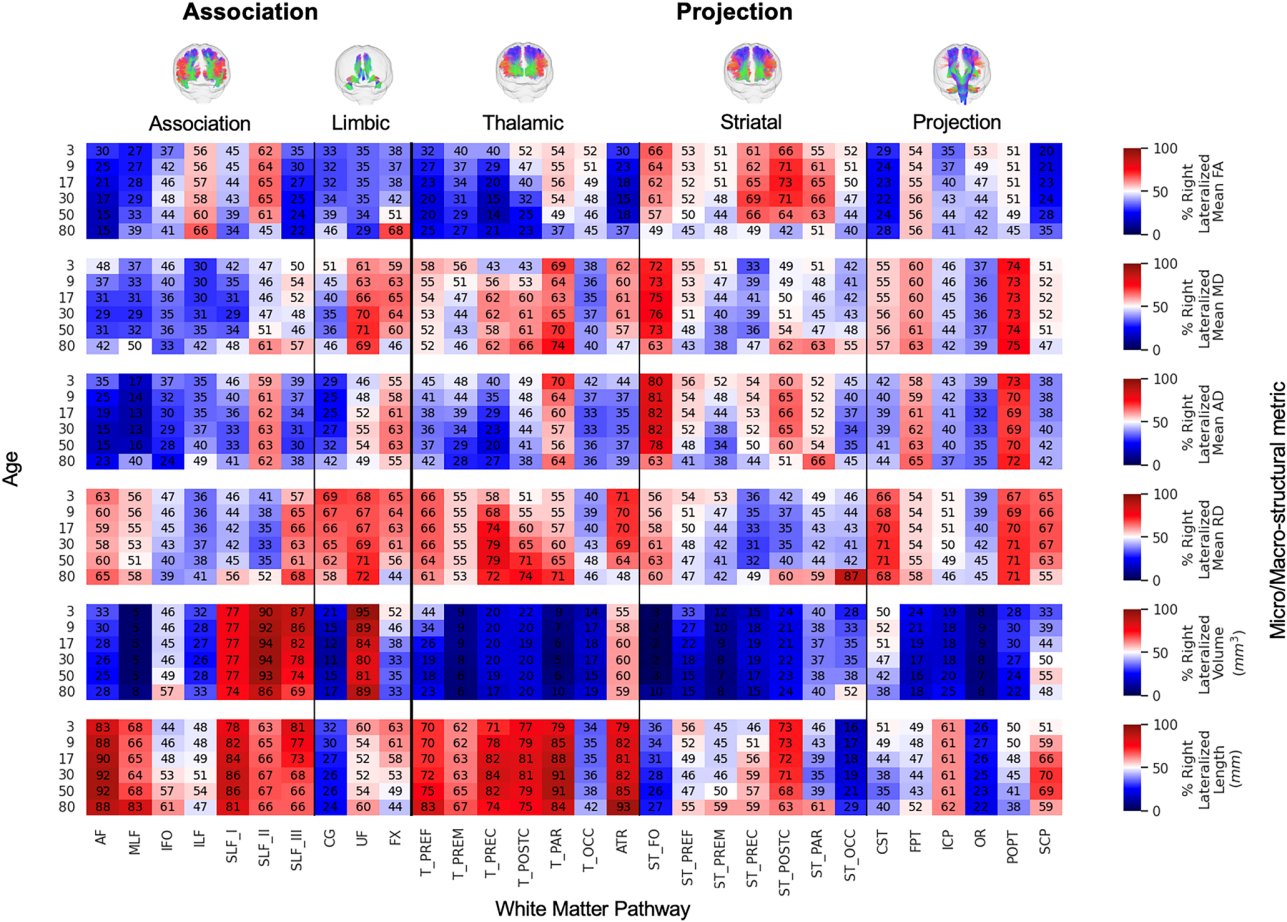
Population prevalence of rightward asymmetry across individuals. This figure complements Figure 4 by showing the percentage of the population that is right‐lateralized for the same features, tracts, and age points. Numerical values and the color scale indicate this prevalence (red: > 50% right‐lateralized; blue: < 50% right‐lateralized, that is, majority left‐lateralized). This visualization reveals the consistency of asymmetry across the population, demonstrating that even for tracts with a small median LI, a strong majority of individuals may share the same direction of lateralization.

To characterize asymmetry at discrete life stages, we examined the population median LI (Figure 4) and the percentage of individuals with rightward lateralization (Figure 5) at six key ages: 3, 9, 17, 30, 50, and 80 years (representative of early childhood, childhood, adolescence, early adulthood, midlife, and older adulthood, respectively). Together, the two figures convey both the typical direction/magnitude (median LI) and the population consensus (prevalence) for each tract–feature–age combination.

The results highlight a considerable range in the degree of lateralization across the connectome. Some pathway‐feature combinations showed a near‐even split in directional preference across the population (i.e., median LI near 0 in Figure 4 and % right‐lateralized near 50% in Figure 5). Others exhibited strong and consistent lateralization, with 75%–90% or more of individuals showing asymmetry in the same direction (e.g., AF, middle longitudinal fasciculus [MLF], and striato‐fronto‐orbital [ST_FO] volume). Importantly, many of these distinct patterns were already clearly established in early childhood (age 3 years).

Examining trends by feature type revealed broad patterns. For microstructure, FA was typically left‐lateralized (negative LIs) across the population for most association and thalamic tracts, with the main exception being the second branch of the fronto‐parietal superior longitudinal fasciculus (SLF II), which tended to be right‐dominant. In contrast, FA in striatal tracts was more often right‐lateralized in ~50%–70% of the population. Other diffusivity measures (MD and AD) showed a modest but consistent leftward asymmetry across many pathways. For macrostructure, tract volume asymmetries were highly heterogeneous. Strong leftward lateralization (negative LIs) was evident for the AF (~75% of population), MLF (~95% of population), ST_FO (> 95% of population), optic radiation (OR, > 90% of population), and the cingulum bundle (CG; > 80%). Strong rightward asymmetry (positive LIs) was observed for all branches (I, II, and III) of the fronto‐parietal superior longitudinal fasciculus (SLF_I, 74%–77%; SLF_II, 86%–94%; and SLF_III, 66%–81%), and the uncinate fasciculus (UF, 80%–95%). Length also varied considerably; for instance, the length of AF was right‐lateralized despite volume being left‐lateralized, whereas length of CG was left‐lateralized alongside its volume. Visual inspection of the heatmaps suggests these patterns are further modulated by age, a dynamic further explored in the final section.

### 3.3 Do Asymmetries Reverse Direction Across the Lifespan?

**FIGURE 6:**
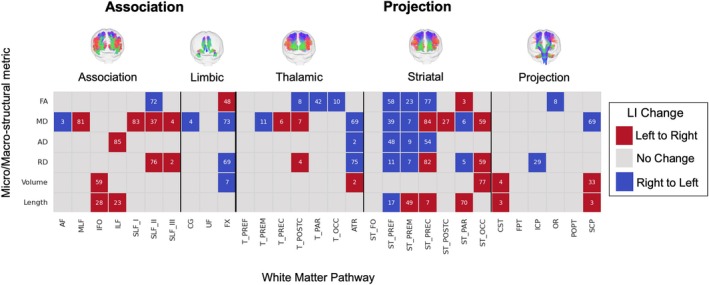
Lifespan trajectories reveal dynamic reversals in the direction of hemispheric asymmetry. Each cell indicates a “lateralization reversal,” where the population‐median LI for a given tract‐feature combination crosses the zero line. Red cells indicate a transition from leftward to rightward asymmetry (negative to positive LI), while blue cells indicate a transition from rightward to leftward (positive to negative LI). The overlaid numbers represent the age (in years) at which this reversal occurs. Grey cells indicate that lateralization reversal did not occur. The results demonstrate that while reversals are sparse, they occur across all major pathway types and at various life stages.

Our analysis of the population median LI trajectories identified instances of “lateralization reversal,” where the typical direction of (population‐level) asymmetry shifts from one hemisphere to another across the lifespan (Figure 6). This indicates that hemispheric lateralization is not a fixed characteristic but can dynamically evolve with age. As shown in the figure, reversals in the median LI, though relatively sparse, are evident across all major tract groups. There are few patterns that generalize across all pathways or all features, although we note FA tends to reverse from right to left (if this occurs), whereas diffusivity and macrostructural measures generally show the opposite left to right trend. The timing of these population‐level transitions varies considerably across the connectome. For instance, some median LIs reversed direction early in development, while others did so much later in life. This heterogeneity suggests complex and varying degrees of hemispheric plasticity over the lifespan, likely reflecting distinct underlying neurodevelopmental and neurodegenerative processes.

### 3.4 How Does the Magnitude of Asymmetry Evolve During Different Life Stages?

**FIGURE 7:**
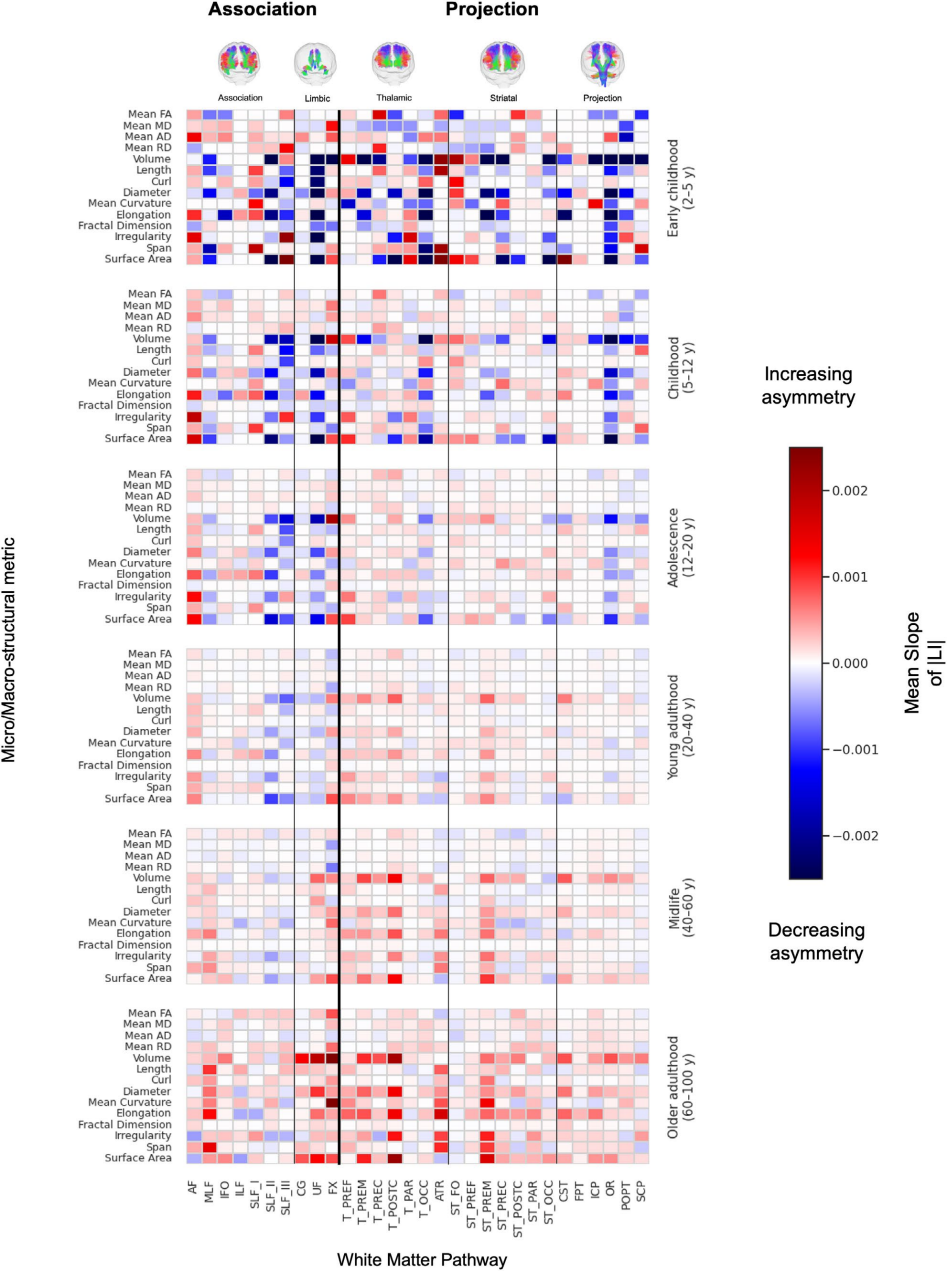
The evolution of asymmetry is a dynamic and age‐specific process. This figure shows the mean rate of change (slope) of the population‐median absolute lateralization index (|LI|) within six distinct life stages (rows). Each cell represents the slope for a given feature and white matter tract. Supplementary macrostructural features were analyzed for exploratory characterization. Warmer colors (red) indicate an increase in the magnitude of asymmetry (strengthening of lateralization), while cooler colors (blue) denote a decrease in the magnitude of asymmetry (attenuation of lateralization). Childhood shows heterogeneous increases and decreases across pathways, adolescence largely continues these trends with smaller slopes, young adulthood is comparatively muted, and later adulthood shows a predominant increase—most marked for macrostructural measures (volume, surface area).

Finally, we quantified how the magnitude of asymmetry evolves by calculating its rate of change (the slope of the absolute value of the median LI) within six distinct life stages: early childhood (2–5 years), childhood (5–12 years), adolescence (12–20 years), early adulthood (20–40 years), midlife (40–60 years), and older adulthood (60–100 years). Figure 7 shows these results, where an increasing asymmetry (greater |LI|, meaning *more* leftward or *more* rightward asymmetry) is indicated in red, and decreasing asymmetry (convergence toward symmetry, that is, *less* leftward or *less* rightward) is shown in blue.

Childhood shows the most heterogeneous patterns. Within the same feature class, some pathways show increasing symmetry while others show decreasing. For volume, increasing asymmetry is observed in AF, inferior longitudinal fasciculus (ILF), and several frontal/prefrontal connections (e.g., thalamo‐prefrontal [T_PREF], ATR, FPT), whereas SLF‐II, SLF‐III, UF, and OR tend to decrease. For FA, increases appear in AF, SLF_III, and thalamo‐premotor (T_PREM), while ST_FO, MLF, and ILF decrease. This mixture suggests active, pathway‐specific refinement in childhood.

Adolescence largely continues the childhood directions but with smaller slopes (i.e., changes persist but are less pronounced), consistent with a tapering of developmental remodeling. Young adulthood shows mostly modest shifts, with residual increases in selected macrostructural measures (volume and surface area standing out) and relatively small changes in diffusion metrics.

In middle/late adulthood, a trend emerges where the magnitude of asymmetry generally increases across a wide range of tracts and features. This effect was particularly strong for macrostructural measures like volume and surface area, which had generally been tending towards symmetry in earlier stages of development. For example, the MLF, UF, and nearly all projection pathways showed a strengthening of asymmetry in later life. In contrast, decreases in asymmetry (i.e., tracts becoming more symmetric) were less frequent, though notable exceptions were observed (including the volume of the SLF_II and SLF_III).

## 4 Discussion

This study represents the most comprehensive investigation of white matter asymmetry across the human lifespan. By leveraging a large‐scale dataset of over 35,000 individuals from 50 neuroimaging cohorts, we generated normative lifespan trajectories for 6 distinct microstructural and macrostructural features across 30 lateralized long‐range white matter pathways. Our principal findings reveal a complex and dynamic pattern of brain asymmetry across pathways, features, and the lifespan. First, we demonstrate that white matter asymmetries are widespread, affecting nearly all studied pathways, though the magnitude and lateralization vary considerably. Second, our results show that these asymmetries are feature‐dependent, with different or even diverging patterns observed between measures of tissue microstructure and those reflecting pathway macrostructure. Third, we highlight that asymmetry profiles are pathway specific, likely reflecting the unique functional roles and developmental timelines of different neural pathways. Finally, we show that these asymmetries are not static but are highly dynamic, characterized by rapid, heterogeneous changes during childhood and a general trend of becoming more pronounced during the aging process. Together, these lifespan charts quantify the magnitude, direction, and age‐specific variability of white matter asymmetry, providing a reference to interpret individual differences and to study deviations in development, aging, and disease.

### 4.1 Widespread White Matter Asymmetry and Comparison With Prior Literature

Our study confirms and substantially extends the view of hemispheric asymmetry as a fundamental organizing principle of the brain's white matter connectome. By charting 30 pathways across the lifespan, we establish that asymmetries are common, yet heterogeneous. A key insight from our normative modeling approach is the distinction between the *magnitude* of an asymmetry and its *consistency* across the population. While microstructural asymmetries often had modest population‐median LIs, our centile charts reveal that even a small median effect can reflect a strong population consensus, with 70%–90% of individuals showing the same directional asymmetry (Figure 3). Conversely, a larger median LI did not always guarantee unanimity. This highlights a critical limitation of relying on mean‐based analyses alone and underscores the power of a distributional perspective for understanding brain lateralization.

These analyses also highlight that the nature and degree of asymmetry differ across both structural features and anatomical pathways, with many of these distinct lateralization patterns already evident in early infancy. Our lifespan perspective also revealed that inter‐individual variability in asymmetry is not uniform (Figure 3, centile bands), with the widest centile bands observed in early childhood and in older age. This pattern likely reflects heterogeneous maturational rates and synaptic pruning during development (Dubois et al. 2014; Dubois et al. 2025), and the cumulative impact of diverse genetic, environmental, and health‐related factors during aging (Habeck et al. 2017).

Compared with prior reports studying asymmetry—typically smaller, age‐restricted, and focused on a few tracts or metrics (Table 2, for a review of sample size and age range see Table S3)—our lifespan curves generally corroborate well‐established findings, such as the leftward FA and volumetric asymmetry of the arcuate fasciculus in adulthood, validating our large‐scale harmonization. However, our data also help clarify previously conflicting reports in the literature (Dulyan et al. 2025)—where some studies demonstrated structural features to be left‐lateralized and others demonstrated those same features to be right‐lateralized—particularly for association pathways like the inferior fronto‐occipital fasciculus (IFOF) and UF. For the IFOF, some studies report volume to be left‐lateralized (Powell et al. 2006), whereas others (Wu et al. 2016) (and our results) suggest there is essentially no lateralization of IFOF volume. Similarly, for the UF, some studies have demonstrated FA to be right‐lateralized (Parekh et al. 2023), whereas others (Hasan et al. 2009; Powell et al. 2006) (and our results) indicate leftward lateralization. For the CST, some studies report left‐lateralized microstructure (Angstmann et al. 2016; Demnitz et al. 2021; Parekh et al. 2023), whereas others (Parekh et al. 2023; Schmithorst et al. 2008) (and our results) indicate right‐lateralized microstructure with left‐lateralized macrostructure—differences that likely reflect metric choice and age window.

**TABLE 2:** Comparison of our findings and those previously demonstrated in the literature. Previously demonstrated findings are presented as [Tract]‐[Feature]‐[Lateralization] (additional qualifiers).

Study	Age range	Findings	Our findings	Study	Age range	Findings	Our findings	Study	Age range	Findings	Our findings
Amemiya et al. (2021)	6–81 years	SLF III‐FA‐R	**✗**	Parekh et al. (2023)	8–12 years	ACR‐AD‐R		Thiebaut de Schotten, Dell'Acqua, et al. (2011) and Thiebaut de Schotten, Ffytche, et al. (2011)	18–22 years	CST‐volume‐L	**✓**
		SLF II‐FA‐R	**✓**			ALIC‐FA‐R				CST‐streamlines‐L	
		SLF I‐qR1‐L				ALIC‐AD‐R				OR‐FA‐R	**✗**
Budisavljevic et al. (2021)	18–29 years	SLF‐degree‐L (right‐handers)				ALIC‐MD‐R				ILF–FA–L	**✓**
		SLF‐degree‐more symmetric (left‐handers)				CG‐FA‐L	**✓**			IFOF–streamlines–R	
Gong et al. (2005)	20–40 years	CG‐FA‐L	**✓**			CG‐AD‐L	**✓**			AF‐volume‐R	**✗**
Schmithorst et al. (2008)	5–18 years	Splenium‐FA‐L (girls)				CG‐MD‐R	**✗**			AF_ant–volume–R	
		AF‐FA‐R (boys)	**✗**			CG‐RD‐R	**✓**			AF_ant‐streamlines‐R	
		Frontal WM‐FA‐R (boys)				CP‐FA‐ R				AF_ant‐FA‐R	
		Occipito‐parietal WM‐FA‐R (boys)				CP‐AD‐R				AF_long–volume–L	
		CST‐MD‐R (boys)	**✓**			CP‐MD‐R				AF_long‐streamlines‐L	
		AF‐MD‐R (girls)	**✗**			CST‐FA‐L	**✗**	Vernooij et al. (2007)	25–54 years	AF‐RFD‐L	
		Occipito‐parietal WM‐MD‐R (girls)				CST‐MD‐R	**✓**	Wiberg et al. (2019)	8–17 years	CG‐FA‐L	**✓**
Büchel et al. (2004)	21–43 years	AF–FA–L	**✓**			CST‐RD‐R	**✓**			Post IC‐FA‐L	
		CC–FA–R				EC‐FA‐L				Thalamus‐FA‐L	
		PreCG–FA–Contralateral to dominant hand	**✓**			EC‐AD‐R				Frontal WM‐FA‐L (males)	
Catani et al. (2007)	18–22 years	AF‐FA‐L	**✓**			EC‐MD‐R				Frontal WM‐FA‐R (females)	
Dayan et al. (2015)	5–18 years	OR‐Volume‐L	**✓**			EC‐RD‐R				Ant IC‐FA‐L (males)	
		OR‐RD‐L	**✓**			FX‐AD‐R	**✗**			Ant IC‐FA‐~R (females)	
		OR‐MD‐L	**✓**			FX‐MD‐R	**✗**	Shu et al. (2021)	4–12 years	ATR‐FA‐R	**✗**
		OR‐AD‐L	**✓**			FX‐RD‐R	**✓**			CST‐FA‐R	**✓**
		OR‐FA‐L (girls>boys)	**✓**			ICP‐FA‐R				IFOF‐FA‐L	**✓**
Demnitz et al. (2021)	62–70 years	CST‐FA‐L	**✗**	Parekh et al. (2023)	8–12 years	ICP‐RD‐L		Shu et al.(2021)	4‐12 years	AF‐FA‐L	**✓**
		CST‐MD‐L	**✗**			ML‐FA‐L		Dubois et al. (2016)	6–22 weeks 21–27 years	AF‐FA‐L (infants)	**✓**
Honnedevasthana Arun et al. (2021)	22–35 years	SLF_I–FD–L				ML‐AD‐R				SLF‐FA‐L (infants)	**✓**
		SLF_II–FD–L				ML‐MD‐R				MLF‐FA‐L (infants)	**✓**
		SLF_IV–FD–R				ML‐RD‐R				AF‐AD‐L (infants)	**✓**
		CG–FD–L				PCR‐AD‐R				SLF‐AD‐L (infants)	**✗**
		IFOF–FD–R				PCR‐MD‐R				UF‐AD‐R (infants)	**✗**
		UF–FC–R				PCR‐RD‐R				SLF‐RD‐R (infants)	**✓**
		ILF–FD–L				PLIC‐FA‐L				MLF‐RD‐R (infants)	**✓**
		OR–FD–L				PLIC‐AD‐R				ILF‐RD‐L (infants)	**✓**
		ICa–FD–R				PLIC‐MD‐R				IFO‐RD‐L (infants)	**✓**
		ICp–FD–L				PLIC‐RD‐R		Takao et al. (2013)	25–85 years	AF‐Volume‐L	**✓**
		SFOF–FA–R				PTR‐MD‐R	**✗**			CG‐Volume‐L	**✓**
		ST–FA–R				PTR‐RD‐R	**✓**			CC_genu‐Volume‐L	
		SCR–FA–L				RLIC‐FA‐L				IC‐Volume‐L	
Kumpulainen et al. (2023)	0–5 years	PTR‐FA‐L (infants)	**✓**			RLIC‐AD‐R				SLF‐Volume‐L	**✗**
		OR‐FA‐L (infants)	**✓**			RLIC‐MD‐R				EC‐Volume‐R	
		PCR‐FA‐L (infants)				RLIC‐RD‐R				AF‐FA‐L	**✓**
		UNC‐FA‐R (infants)				SCP‐FA‐L	**✓**			CG_anterior‐FA‐L	**✓**
		PLIC‐FA‐R (infants)				SCP‐AD‐R	**✗**			SLF‐FA‐L	**✓**
		ALIC‐FA‐R (infants)				SCP‐MD‐R	**✗**	Lebel et al. (2019)	4–33 years	AF‐FA‐L	**✓**
		SCR‐FA‐R (infants)				SCP‐RD‐R	**✗**			IC‐FA‐L	
		CG‐FA‐L (5 year‐olds)	**✓**			SCR‐FA‐L				OR‐FA‐L	**✓**
		UF‐FA‐L (5 year‐olds)	**✓**			SCR‐MD‐R				CG‐FA‐L	**✓**
		EC‐FA‐L (5 year‐olds)				SCR‐RD‐R				SLF‐FA‐L	**✓**
		SCR‐FA‐L (5 year‐olds)				SFO‐AD‐R				SCP‐FA‐L	**✓**
		ALIC‐FA‐R (5 year‐olds)				SFO‐MD‐R				UF‐FA‐L	**✓**
		SFOF‐FA‐R (5 year‐olds)				SLF‐FA‐L	**✓**			Frontal‐FA‐R	
		ACR‐FA‐R (5 year‐olds)				SLF‐AD‐R	**✗**			TR‐FA‐R	
Glasser and Rilling (2008)	18–50 years	AF‐FA‐L	**✓**	Parekh et al. (2023)	8–12 years	SLF‐MD‐R	**✗**	Lebel et al. (2019)	4–33 years	Frontal‐FA‐L(child) ‐> R(adolescent)	
Powell et al. (2006)	23–50 years	SLF‐FA‐L	**✓**			SLF‐RD‐R	**✗**	Baudo et al. (2022)	20–40 years	AC_anterior‐fiber projections‐L	
		SLF‐Volume‐L	**✗**			SS‐AD‐R				AC_posterior‐fiber projections‐R	
		IFO‐FA‐L	**✓**			SS‐MD‐R		Briggs et al. (2020)	Not noted	FAT‐volume‐no asymmetry	
		IFO‐Volume‐L	**✗**			SS‐RD‐R				IFO‐volume‐no asymmetry	**✓**
		UF‐FA‐L	**✓**			UF‐FA‐R	**✗**			CG‐volume‐no asymmetry	**✗**
		UF‐Volume‐L	**✗**			UF‐AD‐R	**✗**	Ford et al. (2023)	0–1 years	AF‐FA‐L (1–3.5 months)	**✓**
Takao et al. (2013)	21–29 years	CC_anterior‐FA‐L				UF‐RD‐L	**✗**			ATR‐FA‐R (3–6 months)	**✓**
		CG‐FA‐L	**✓**	Putnam et al. (2010)	24–39 years	Splenium‐FA‐R				UF‐FA‐R (0–1.5 months)	**✗**
		OR‐FA‐L	**✓**	Sboto‐Frankenstein et al. (2014)	19–30 years	FX‐FA‐L	**✓**			Fx‐FA‐L (5–6 months)	**✓**
		SCP‐FA‐L	**✓**	Shu et al. (2015)	23.4 ± 3.7 years 12–35 years (using 3 standard deviations)	AF‐FA‐L	**✓**			IFO‐FA‐L	**✓**
		IC_anterior‐FA‐R				ILF‐FA‐L	**✓**			ILF‐FA‐R	**✓**
		UF‐FA‐R	**✗**			CB‐FA‐L				M1‐FA‐L	
		AF_superior‐FA‐R	**✗**			IFO‐FA‐L	**✓**			S1‐FA‐R	
		AF_inferior‐FA‐R	**✗**			CB‐Fiber Number‐L				CG‐FA‐no asymmetry	**✗**
Thiebaut de Schotten, Dell'Acqua, et al. (2011) and Thiebaut de Schotten, Ffytche, et al. (2011)	Not noted	SLF_I‐Volume‐None	**✗**			OR‐Fiber Number‐R		Hau et al. (2017)	22–34 years	UF_C1‐volume‐L	**✗**
		SLF_II‐Volume‐R	**✓**			ILF‐Fiber Number‐L				UF_C3‐volume‐R	**✓**
		SLF_III‐Volume‐R	**✓**			AF‐Fiber Number‐L				UF_C3‐streamline count‐R	
Hasan et al. (2009)	6–68 years	UF‐FA‐L	**✓**			UF‐Fiber Number‐R				UF_C5‐volume‐R	**✓**
		UF‐AD‐L	**✓**	Wu et al. (2016)	23–40 years	IFO‐Volume‐no asymmetry	**✓**	Panesar et al. (2019)	23–35 years	vTPAT‐connectivity‐L	
Lebel et al. (2019)	5–30 years	AF–FA‐L	**✓**	Angstmann et al. (2016)	11–16 years	CST‐FA‐L	**✗**			dTPAT‐connectivity‐R	
		AF‐Streamlines‐L		Barrick et al. (2007)	20–39 years	AF–FA‐L	**✓**			AF–volume‐L	**✓**
Dalamagkas et al. (2020)	22–35 years	CST‐FA‐no asymmetry	**✗**	Silk et al. (2016)	10–18 years	Caudate–VLPFC‐volume‐R		Panesar et al. (2019)	23–35 years	SLF‐volume‐R	**✓**
		HMFT‐FA‐R				Caudate–DLPFC‐volume‐R					

*Note:* ✓ = agreement between our findings and those presented in the literature. X = disagreement between our findings and those presented in the literature. Grey in “Our Findings” = tract or measure not measured in our work. Grey in “Our Findings” + ✓/X = tract represented in our data as conglomerate or as multiple tracts, though still allowing for gross comparison. Blue in the table is included solely to enhance readability. In cases where tract abbreviations presented in the literature differed from those of our tracts, we presented the tract name using our abbreviations.

Several factors explain discrepancies and the additional patterns detected here. First is scale: with *N* = 35,120, our centile modeling estimates population trajectories rather than relying on small‐sample inference. This makes our study particularly powerful compared to smaller studies and enables its future use as a reference for lifespan asymmetry in healthy patients. Indeed, a major motivation for using a GAMLSS‐based normative framework was its ability to estimate age‐varying distributions while explicitly accounting for between‐cohort heterogeneity (e.g., scanner/protocol differences) within a single model (Rigby and Stasinopoulos 2005). Second, continuous age modeling (rather than single time points or assumption of homogeneity across age) exposes direction changes and non‐linear trends that limited‐age designs miss. Third, tract definition may vary across studies (Schilling, Rheault, Petit, Hansen, et al. 2021). Automated, standardized segmentation (e.g., TractSeg) (Wasserthal et al. 2018) reduces protocol variance but will not replicate every manual or atlas‐based definition used previously. Finally, is the choice of features, as micro‐ and macrostructural features measure different aspects of pathway biology and can often diverge. By providing comprehensive, openly available lifespan asymmetry charts, our work offers a stable reference point to help reconcile these inconsistencies and guide future research.

We additionally generated handedness‐specific lifespan charts in the subset of cohorts with handedness information available (12 cohorts; *N* = 14,220; analyses focused on right‐ vs. left‐handed individuals). Across exemplar tracts and features, handedness‐related differences in the modeled median LI were consistently small (Cohen's *d* [calculated at every integer age 1–80 and averaged across the lifespan] typically < 0.08 and often < 0.02; Figure S1), indicating that handedness explains little additional variance in tract‐level asymmetry in these data. This analysis is necessarily more weakly constrained than our primary models (i.e., does not strictly align with Figure 3) because handedness was unavailable for most cohorts and the left‐handed subgroup was smaller and not evenly distributed across the lifespan—factors known to reduce precision when estimating centile curves within subgroups (i.e., optimally designed growth reference studies often require very large samples per group (Cole 2021)). Despite this, the minimal handedness effects observed here are broadly consistent with prior reports that structural asymmetry relates only weakly to handedness (Powell et al. 2012; Groen et al. 2013; Guadalupe et al. 2014; Kong et al. 2018), even as functional lateralization and language‐network connectivity can differ by handedness (Wiberg et al. 2019).

### 4.2 Divergent Asymmetries of Microstructural and Macrostructural Features

Our findings demonstrate that white matter asymmetry is not “all encompassing” within a given pathway. Rather, it is highly feature dependent, meaning a tract broadly described as “left‐lateralized” may not show leftward “dominance” for all its micro‐ and macrostructural characteristics. For instance, a pathway may exhibit leftward asymmetry for FA and other diffusivities (MD/AD) yet display rightward asymmetry for RD, as is seen in the arcuate fasciculus (Figure 3); or show leftward asymmetry in FA while its overall volume is greater in the right. These examples are widespread throughout our investigation (Figures 4 and 5) and highlight the requirement for specifying which feature (reflecting distinct biological properties) is lateralized. Indeed, diffusion tensor metrics like FA and RD are sensitive to processes like myelination and axonal packing, AD to axonal structure and coherence, and MD to overall water diffusivity, each providing unique insights into pathways' structure (Beaulieu 2002). The field must continue to investigate the biological and functional significance of these feature‐specific asymmetries to better understand brain lateralization.

This divergence is biologically plausible and offers insight into brain organization on multiple scales. Macrostructural asymmetries could represent an earlier, more genetically‐programmed aspect of development that establishes the gross architecture of a pathway (Budisavljevic et al. 2021; Habeck et al. 2017). In contrast, microstructural asymmetries might reflect more dynamic, experience‐dependent processes, such as the activity‐dependent myelination that refines and optimizes a circuit for a specific function (Groen et al. 2013; Powell et al. 2012). This dissociation underscores why simplistic labels like a “dominant” hemisphere or generalized statements about “white matter integrity” can be misleading (Kong et al. 2018). Interpreting lateralization and its functional or dysfunctional implications requires considering both large‐scale architecture and cellular‐level microstructure of white matter pathways.

Diffusion tensor indices (FA, MD, AD, and RD) should not be interpreted as independent biological measures, as they are different summaries of the same tensor model (Wheeler‐Kingshott and Cercignani 2009). In particular, FA is mathematically determined by the tensor eigenvalues and therefore depends on the relative behavior of axial and radial diffusivity. As a result, cases where FA laterality differs from RD (or AD) laterality should be interpreted as complementary descriptors of how diffusion parallel versus perpendicular to the principal fiber direction differs between hemispheres, rather than as evidence for unrelated or competing biological processes.

At the same time, diffusion‐derived metrics are sensitive but not specific to myelin or any single microstructural substrate; FA for example, reflects not only myelin but also broader features of tissue microstructure and organization (e.g., membrane/axon packing, caliber, and fiber geometry) (Beaulieu 2002), and—like all tensor‐derived indices—its interpretation can be biased in voxels with complex fiber architecture (e.g., crossing/branching bundles) and partial‐volume with other tissues (Pierpaoli et al. 1996). For this reason, diffusion‐based laterality is usefully interpreted alongside complementary contrasts that more directly index myelination. As an example, myelin water fraction imaging provides a more myelin‐specific estimate with strong histological correspondence and has also demonstrated hemispheric asymmetries in healthy participants and early development (Deoni et al. 2012), including associations with behavior (reading, language) (Beaulieu et al. 2020; O'Muircheartaigh et al. 2013) and genetics (Ocklenburg et al. 2019). Together, these findings support the view that a subset of the lifespan asymmetry patterns observed here—particularly those involving diffusivity‐based measures—may partly reflect hemispheric differences in myelination, while motivating future multimodal studies that directly combine diffusion modeling with quantitative myelin imaging or biophysical modeling (Novikov et al. 2018).

### 4.3 Pathway‐Specific Patterns of White Matter Asymmetry

While previous work has generated such charts for grey matter (Bethlehem et al. 2022) and white matter (Kim et al. 2025), this is the first study to specifically chart tract‐level asymmetry by creating population‐level curves (describing the population distribution) across age. From a basic neuroscience perspective, this resource helps quantify how neural circuits differ between hemispheres, supporting ongoing efforts to relate large‐scale neuroanatomical organization to functional specialization (Forkel et al. 2022).

Across pathways, we found that asymmetry profiles are tract‐specific and align with several recurring themes described in prior literature. For example, as noted previously, the arcuate fasciculus shows a robust leftward bias for several features across much of the lifespan, consistent with the tract's central role in the dorsal language system. In contrast, frontoparietal association pathways (most notably SLF I, II, and III) show strong rightward macrostructural asymmetry in a large majority (70%–85%) of individuals (Figure 5), a pattern that has been linked to right‐hemisphere dominant attentional/visuospatial networks (Dulyan et al. 2025; Thiebaut de Schotten, Dell'Acqua, et al. 2011), and consistent with right‐lateralized IFOF volume that has been interpreted as a structural basis for the right hemisphere's strengths in visuospatial integration (Thiebaut de Schotten, Ffytche, et al. 2011). Together, these examples (and the widespread asymmetries across all pathways studied) highlight that structural lateralization is not limited to a single “language tract,” but is a distributed property of multiple functional systems. These pathway‐specific reference distributions can help guide future work aiming to link particular asymmetry profiles to individual differences in cognition, behavior, and vulnerability to neurological or psychiatric disorders.

A key point for interpretation is that tract asymmetries often co‐occur with well‐established cortical asymmetries in the same functional systems (Good et al. 2001; Minkova et al. 2017; Ocklenburg et al. 2016; Toga and Thompson 2003), suggesting coordinated hemispheric specialization across gray‐matter nodes and the long‐range pathways that connect them. In the language network, Roll (2024) reports pronounced leftward surface‐area asymmetry in Heschl's gyrus and a dissociation within the inferior frontal gyrus, where pars opercularis shows leftward surface‐area asymmetry while anterior IFG subdivisions show the opposite pattern (Roll 2024). Roll also highlights a broader anatomical signature in left‐hemisphere language regions—larger surface areas paired with thinner cortex and a higher white‐to‐gray matter ratio—a profile consistent with a more myelinated and efficient substrate for rapid categorical processing. These cortical asymmetries provide an anatomical context for the left‐lateralized AF that we observe: the AF is the major long‐range association pathway linking posterior temporal/auditory regions with posterior inferior frontal speech regions, and multiple studies have shown that inter‐individual differences in arcuate laterality relate to functional language lateralization. In our data, this same circuit shows complementary tract‐level asymmetries across much of the lifespan, including leftward asymmetry in AF volume and FA (with a rightward asymmetry in mean streamline length), consistent with hemispheric differences in both pathway architecture and diffusion‐derived tissue organization. These gray and white matter asymmetries likely reflect partly shared developmental and genetic influences, and our lifespan charts provide a starting point for testing how they covary with functional lateralization and behavior across age.

Beyond association pathways, our results reveal that thalamic projection tracts tend to exhibit leftward microstructural asymmetry while striatal projection tracts show the opposite pattern, despite their partial anatomical overlap. This divergence is consistent with prior work demonstrating lateralized, circuit‐specific differences in thalamocortical versus corticostriatal circuitry and likely reflects fundamental organizational differences in their connectivity and functional roles (Foster et al. 2021; Martel and Galvan 2022; Shepherd and Yamawaki 2021). Thalamocortical pathways demonstrate reciprocal and nonreciprocal connections with layer‐specific cortical termination patterns that support hierarchical information flow and sensory integration, whereas corticostriatal pathways exhibit greater convergence and divergence to enable integration of distributed cortical inputs for action selection and motor–cognitive control (Martel and Galvan 2022; Shepherd and Yamawaki 2021; Wolff et al. 2022).

### 4.4 Dynamic Nature of Asymmetry Across the Lifespan

A central finding of this work is that white matter asymmetries are not static but follow dynamic trajectories across the lifespan (Figure 3). These dynamics are evident in both the population median LI trajectories and the corresponding population variability. While the typical magnitude of the median LI for many features, particularly microstructural ones, often remains within a relatively constrained range (e.g., frequently staying below ±0.1), especially during most of adult life, their specific values and trajectories show population‐level changes over decades. This evolution varies considerably by pathway: some tracts exhibit clear shifts and modulations in their median asymmetry profiles across developmental and aging periods (e.g., ATR), while others maintain a more consistent pattern of lateralization and population distribution throughout much of life (e.g., AF). The periods of most pronounced change in these asymmetry trajectories—reflecting significant neural reorganization generally occur during very early development and again in later life during healthy aging. It is important to highlight the cross‐sectional nature of the current dataset; because of this, we cannot (or do not) follow individual “change” but rather cross‐sectional median and ranges across the population.

**FIGURE 8:**
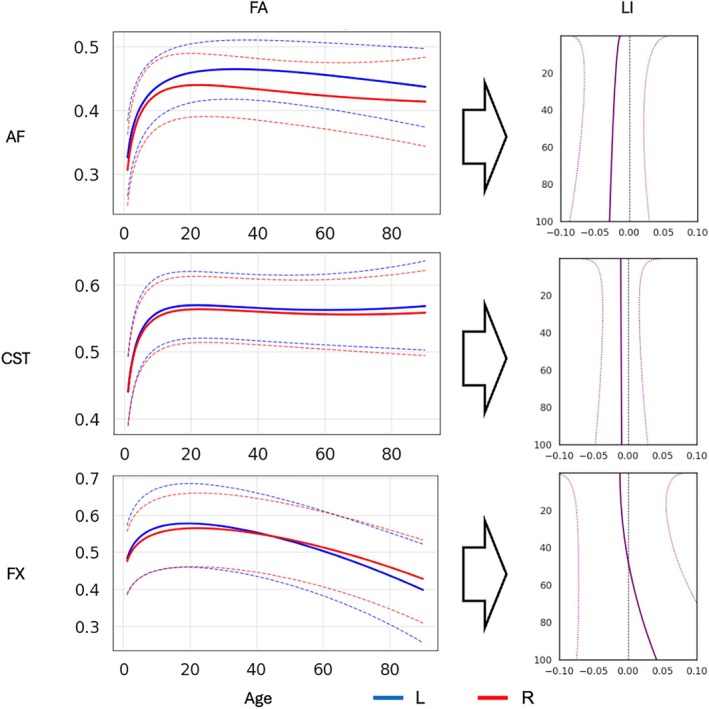
Interpreting LI trajectories in the context of bilateral lifespan trajectories. Left‐ and right‐hemisphere lifespan trajectories for exemplar tract–feature combinations (left panels) are shown alongside the corresponding LI trajectories (right panel). The figure illustrates that larger asymmetries arise when left and right trajectories remain widely separated, smaller asymmetries arise when trajectories are closely aligned, and LI shifts or reversals can occur when hemispheric trajectories change at different rates across age. Importantly, LI curves were estimated by computing LI at the individual level and fitting normative models to those LI values; they are not derived by algebraically differencing the plotted left/right population trajectories.

Interpreting lifespan trajectories of the lateralization index (LI) requires considering the underlying bilateral trajectories from which LI is computed. Because LI is a normalized contrast of left‐ and right‐hemisphere tract properties, the same apparent change in LI (e.g., attenuation or strengthening) can arise through multiple mechanisms: a decrease in the feature on the initially dominant side, an increase on the contralateral side, or differences in the timing and slope of change across hemispheres. This is illustrated in Figure 8, which juxtaposes exemplar left‐ and right‐hemisphere lifespan curves for tract microstructure with the corresponding LI trajectory. Large, stable LIs occur when the two hemispheric trajectories remain separated across age (e.g., AF), whereas small LIs occur when trajectories track closely (e.g., CST). In other cases, LI shifts or reversals can emerge when left and right pathways evolve at different rates such that the trajectories converge, diverge, or cross (e.g., FX). Together, this reinforces that white matter asymmetry is an emergent property of paired hemispheric development and aging, rather than a change attributable to one hemisphere alone.

This interpretation is consistent with prior diffusion MRI lifespan studies (Beck et al. 2021; Kim et al. 2025; Lebel et al. 2012; Schilling et al. 2023; Villalón‐Reina et al. 2024; Zhu et al. 2025) showing that white matter microstructure follows tract‐specific, often non‐linear developmental and aging trajectories rather than a single uniform pattern across contralateral pathways or different pathways.

We also demonstrate, for the first time to our knowledge, the presence of “lateralization reversal”, where the typical direction of asymmetry for a given feature shifts across the lifespan. This phenomenon occurred in almost every tract we measured and for at least one, and often more, measures of connectivity (Figure 6), although the overall frequency was indeed sparse across pathways and features. While previous studies have demonstrated patterns of development of asymmetry over short age ranges (Ford et al. 2023; Kumpulainen 2024; Lebel et al. 2019), identifying specific reversal points is possible by including data across the entire lifespan. The neurodevelopmental and clinical significance of this phenomenon is, to our knowledge, completely unexplored.

Finally, our analysis reveals another novel lifespan trend: the degree of white matter asymmetry shows a general tendency to increase from mid‐life to old age (Figure 7). While asymmetry in infancy/development has been a focus of prior research (Dubois et al. 2016), its evolution during aging is less understood with limited prior investigations (Demnitz et al. 2021). Our results demonstrate that several features (volume, diameter, elongation, irregularity, and surface area) become more asymmetric with age across most pathways, and many other features show similar trends. We hypothesize that these findings are reflective of natural age‐related neurodegenerative processes—occurring in individuals over 50 years old—where one hemisphere's pathways (or specific aspects of their structure) are more vulnerable or decline at a faster rate than their contralateral counterpart, leading to the amplification of initially subtle asymmetries. Future studies are crucial to determine the neurocognitive and clinical impact, if any, of these age‐related increases in white matter asymmetry.

### 4.5 Functional Implications and Clinical Relevance of White Matter Asymmetry

Our findings also carry potential clinical implications. As research accumulates, white matter asymmetry measures could serve as biomarkers for certain conditions such as schizophrenia (Miyata et al. 2012) and Parkinson's disease (Zhu et al. 2023) or as indices of typical vs. atypical brain development, such as in autism spectrum disorder (Joseph et al. 2014). Asymmetry is a particularly attractive biomarker since it is inherently normalized to the individual via its computation as ratios or differences within the same scan. In children with neurodevelopmental disorders, it has been demonstrated that lateralization indices of diffusion metrics might serve as clinically useful imaging biomarkers, helping to detect sensory processing dysfunction with less confounding variance than absolute measures (Parekh et al. 2023). In neurodegenerative diseases, asymmetry often manifests as an asymmetrical onset of pathology. Parkinson's disease typically begins with unilateral motor deficits (reflecting greater degeneration of one side's nigrostriatal pathway), and patients correspondingly show asymmetric cortical changes in several hemispheric regions (Claassen et al. 2016); similar investigations into asymmetrical changes in white matter structural have begun to be conducted (Zhu et al. 2023; Li et al. 2026). With continued development, detection of such asymmetry measures can complement other markers to improve early detection of atypical brain development or pathology.

The role of asymmetry across disease states has only begun to be characterized. When considering clinical populations, white matter asymmetry has been a topic of intense investigation. Research in autism, for example, has observed that typical lateralization patterns are often reduced or altered. Children with autism spectrum disorder have been shown to have significantly diminished asymmetry of white matter microstructure, with many of the normal left–right differences in FA, AD, and RD being smaller in magnitude in autism spectrum disorder, indicating a more symmetrical white matter organization (Carper et al. 2016; Mundorf et al. 2021). Schizophrenia is another condition where white matter asymmetry is studied: some patients show a reduction or reversal of asymmetry in the AF and SLF, among others, with reduced asymmetry in the FA being seen in patients experiencing auditory hallucinations (Catani et al. 2011), possibly underlying the disturbed lateralization of language processing in schizophrenia (Mundorf et al. 2021). In such investigations of pathology‐associated asymmetries, our work can serve as a cornerstone for future comparisons. By providing a multitude of tracts and measures modeled across the lifespan, our charts can reasonably be used as healthy controls in any study which seeks to investigate a particular tract, connectivity measure, and/or critical age range in an abnormal brain state. Our findings in healthy controls, therefore, potentially enable the discovery of different asymmetries that arise in pathological states, providing insight into the mechanism of a given disease.

## 5 Limitations

Our study had notable limitations. We note the results for the fornix (FX) should be interpreted with caution as they could be a result of technical tractography artifacts rather than a biological changes; while this is particularly true of FX, such considerations can be grossly applied to all tractography methods. Macrostructural features are susceptible to the methods used to grid data and so can vary between studies. Similarly, different protocols for delineation of pathways can lead to different regions being included or excluded from a given pathway, lending further complexity to the matter of comparing study results. Lastly, there is overlap in the regions which define given pathways, meaning that microstructural measures in those regions are not unique to the given pathway. This is an inherent limitation of tract‐based summaries of voxel‐wise diffusion tensor metrics: even when measures are weighted by streamline density or restricted to a tract mask, the underlying scalar values are still estimated at the voxel level and can reflect signal contributions from other pathways in regions of crossing/overlap (Schilling, Tax, Rheault, Hansen, et al. 2021).

## 6 Conclusion

This study provides the most extensive characterization of white matter asymmetry across the human lifespan to date, generating comprehensive normative trajectories for 6 structural features across 30 distinct white matter pathways in over 35,000 healthy individuals from infancy to old age. Our key findings demonstrate that white matter asymmetries are widespread yet highly specific to pathways and structural features. Furthermore, these asymmetries are not static but exhibit dynamic changes throughout development and aging, including reversals in direction and a general trend of increasing asymmetry magnitude in later life. These lifespan charts of white matter asymmetry offer a resource for advancing our understanding of brain lateralization principles, investigating inter‐individual variability in brain structure, and providing a reference to assess deviations in neurodevelopmental, aging, and clinical populations.

## Funding

This work was supported by National Institutes of Health (K01‐EB032898, K01‐AG073584, 1R01EB017230‐01A1, K24‐AG046373, 1S10OD020154‐01, 1S10OD023680‐01, U01‐AG024904, U24 AG072122, 1S10 OD021771‐01), Vanderbilt University (VR3029, UL1‐TR000445, UL1‐TR002243, VR53419), National Institute on Aging (R01‐AG034962, R01‐AG056534, R01‐AG062826, U24 AG074855, U01 AG068057, R01 AG059716, R01AG017917, P30AG10161, P30AG072975, R01AG022018), Alzheimer's Association (IIRG‐08‐88733), National Institute of Child Health and Human Development (RC2DA029475‐01, R01HD055741‐01, R01 HD089474, R37 HD095519, R01 HD044073, R01 HD067254, P50 HD103537), National Institute of General Medical Sciences (T32GM007347, T32GM152284), Donders Mohrmann Fellowship (2401515), Nederlandse Organisatie voor Wetenschappelijk Onderzoek, National Institute of Mental Health (BRAINS R01MH094639‐01, R01MH129634), and Child Mind Institute.

## Conflicts of Interest

The authors declare no conflicts of interest.

## Supporting information


**Figure S1:** Handedness effects on lifespan white matter asymmetry are small. Handedness‐stratified normative trajectories of the lateralization index (LI) are shown for right‐handed (blue) and left‐handed (red) individuals in the subset of cohorts with handedness data (12 cohorts; *N* = 15,548; ambidextrous individuals excluded from the handedness‐stratified curves). Rows correspond to example tracts (AF, ATR, FPT, CST) and columns to features (FA, MD, AD, RD, tract volume, tract length). Solid lines denote the modeled population median (50th centile) LI as a function of age; dotted lines indicate the normative spread (2.5th and 97.5th centiles). The vertical dashed line marks LI = 0 (symmetry); positive values indicate rightward asymmetry and negative values indicate leftward asymmetry. Panel annotations report Cohen's d, computed as the mean separation between handedness‐specific median LI trajectories across age (standardized by pooled variability), and demonstrate consistently small handedness‐related differences across tract–feature combinations.
**Figure S2:** Age‐related trajectories of lateralization indices across white matter tracts and diffusion/shape measures. Each subplot displays the median (solid lines) and 95% centile interval (dotted lines) of lateralization index (LI) across age for males (blue) and females (red). LI was modeled using the GAMLSS framework with a normal distribution family. Rows represent different microstructural and geometric measures (e.g., FA, MD, volume, and curvature), while columns correspond to specific white matter tracts (e.g., AF, SLF, and CST). Negative values indicate leftward asymmetry; positive values indicate rightward asymmetry. Vertical dashed lines at *x* = 0 highlight the zero‐asymmetry point. Curves are aligned across tracts to enable direct comparison of asymmetry magnitude and lifespan trajectory by sex and measure. We observe asymmetry patterns change across tracts, measure, and age.
**Figure S3:** Age‐related derivatives of the median LI across white matter tracts and measures. Each subplot shows the first derivative of the 50th percentile (median) LI trajectory across age (*y*‐axis, from 1 to 100 years) for both males (blue) and females (red) (visually hard to see as they overlap each other). Columns correspond to four major white matter tracts (AF, ATR, FPT, and CST), and rows correspond to six asymmetry measures: fractional anisotropy (FA), mean diffusivity (MD), axial diffusivity (AD), radial diffusivity (RD), volume, and average streamline length. The vertical dashed line at x = 0 indicates zero change in asymmetry. Derivative curves show strong early developmental changes, especially in diffusion measures, with greater variability in structural metrics and some divergence between sexes.
**Table S1:** Overview of 50 neuroimaging datasets. Including subject, session, and scan totals.
**Table S2:** Acquisition information for dMRI data from each dataset, along with the respective scanning/facility location.

**Table S3:** Review of studies investigating white matter asymmetry at various points in the lifespan. The study method, micro‐ and macro‐structural features investigated, number of participants, age range, and key findings are summarized.

## Data Availability

Population curves for each pathway and each feature are in npy format and available on Zenodo [https://doi.org/10.5281/zenodo.18202413]. Code for GAMLSS fitting is at https://github.com/MASILab/Asymmetry/src/fit_model_pynm.py and https://github.com/MASILab/Asymmetry/src/fit_model_handedness_pynm.py. Code to generate figures is at https://github.com/MASILab/Asymmetry/src/main_text_figures.ipynb, https://github.com/MASILab/Asymmetry/src/handedness_figures.ipynb, and https://github.com/MASILab/Asymmetry/src/dataset_plots.ipynb. Additionally, the GAMLSS models for each pathway and each feature are available on Zenodo.
